# Supplementary material for: Metabolic response of blood vessels to TNFα
Source: eLife. 2020 Aug 4;9:e54754. doi: 10.7554/eLife.54754 (PMC7476757; doi:10.7554/eLife.54754)
Supplement: Supplementary file 1. [file elife-54754-supp1.docx]

**Supplementary File 1**

| Bioactive lipid | PMID |
| --- | --- |
|  |  |
| PGF1α | 3110148 |
| PGF2α | 11438482 \| 6999547 \| 1415588 |
| PGF3α | 16297610 |
| PGE2* | 30112590 |
| PGE1 | 6999547 \| 24431002 \| 12494264 |
| PGD2 | 6999547 \|30734298 \| 29671869 |
| 13, 14-dihydro-PGF2α |  |
| PGA2 | 6999547 \| 15723383 |
| 8-iso-13, 14-dihydro-PGF2α |  |
| 8-iso-PGF2α* | 11344105 \| 15640282 \| 18802021 \| 10711349 |
| 8-iso-PGE2 | 12716476 \| 10711349 \| 24646155 |
| 8-iso-PGE1 | 10711349 |
| 5-iPF2α |  |
| 8, 12-iPF2α IV |  |
| LPA C14:0 | 25825155 \| 30928673 |
| LPA C16:1 | 25825155 \| 30928673 |
| LPA C22:6* | 25825155 \| 30928673 |
| LPA C18:2 | 25825155 \| 30928673 |
| LPA C20:4 | 25825155 \| 30928673 |
| LPA C22:5* | 25825155 \| 30928673 |
| LPA C16:0 | 25825155 \| 30928673 |
| LPA C18:1 | 25825155 \| 30928673 |
| LPA C22:4 | 25825155 \| 30928673 |
| cLPA C20:4 | 25013374 \| 18554524 |
| LPA C18:0 | 25825155 \| 30928673 |
| cLPA C18:2 | 25013374 \| 18554524 |
| cLPA C16:0 | 25013374 \| 18554524 |
| cLPA C18:1 | 25013374 \| 18554524 |
| cLPA C18:0 | 25013374 \| 18554524 |
| S-1-P C18:1 | 28609704 \| 31049553 \| 20577214 \| 27565080 |
| Sph C18:1 | 28609704 \| 31049553 \| 20577214 \| 27565080 |
| Spha C18:0 |  |
| PAF C16:0 | 17588613 \| 12038971 |

*Validated markers of oxidative stress.
