# Supplementary material for: Metabolic response of blood vessels to TNFα
Source: eLife. 2020 Aug 4;9:e54754. doi: 10.7554/eLife.54754 (PMC7476757; doi:10.7554/eLife.54754)
Supplement: Supplementary file 2. [file elife-54754-supp2.docx]

**Supplementary File 2**

|  | Stock concentration (nM) | | | | | | | | |
| --- | --- | --- | --- | --- | --- | --- | --- | --- | --- |
| Bioactive lipid | C9 | C8 | C7 | C6 | C5 | C4 | C3 | C2 | C1 |
| PGF1α | 53.3 | 26.7 | 13.3 | 6.7 | 3.3 | 1.7 | 0.8 | 0.4 | 0.2 |
| PGF2α | 53.3 | 26.7 | 13.3 | 6.7 | 3.3 | 1.7 | 0.8 | 0.4 | 0.2 |
| PGF3α | 53.3 | 26.7 | 13.3 | 6.7 | 3.3 | 1.7 | 0.8 | 0.4 | 0.2 |
| PGE2 | 53.3 | 26.7 | 13.3 | 6.7 | 3.3 | 1.7 | 0.8 | 0.4 | 0.2 |
| PGE1 | 53.3 | 26.7 | 13.3 | 6.7 | 3.3 | 1.7 | 0.8 | 0.4 | 0.2 |
| PGD2 | 53.3 | 26.7 | 13.3 | 6.7 | 3.3 | 1.7 | 0.8 | 0.4 | 0.2 |
| 13, 14-dihydro-PGF2α | 41.9 | 21.0 | 10.5 | 5.2 | 2.6 | 1.3 | 0.7 | 0.3 | 0.2 |
| PGA2 | 53.3 | 26.7 | 13.3 | 6.7 | 3.3 | 1.7 | 0.8 | 0.4 | 0.2 |
| 8-iso-13, 14-dihydro-PGF2α | 53.3 | 26.7 | 13.3 | 6.7 | 3.3 | 1.7 | 0.8 | 0.4 | 0.2 |
| 8-iso-PGF2α | 53.3 | 26.7 | 13.3 | 6.7 | 3.3 | 1.7 | 0.8 | 0.4 | 0.2 |
| 8-iso-PGE2 | 53.3 | 26.7 | 13.3 | 6.7 | 3.3 | 1.7 | 0.8 | 0.4 | 0.2 |
| 8-iso-PGE1 | 53.3 | 26.7 | 13.3 | 6.7 | 3.3 | 1.7 | 0.8 | 0.4 | 0.2 |
| 5-iPF2α | 53.3 | 26.7 | 13.3 | 6.7 | 3.3 | 1.7 | 0.8 | 0.4 | 0.2 |
| 8, 12-iPF2α IV | 53.3 | 26.7 | 13.3 | 6.7 | 3.3 | 1.7 | 0.8 | 0.4 | 0.2 |
| LPA C20:4 | 2400.0 | 1200.0 | 600.0 | 300.0 | 150.0 | 75.0 | 37.5 | 18.8 | 9.4 |
| LPA C16:0 | 2400.0 | 1200.0 | 600.0 | 300.0 | 150.0 | 75.0 | 37.5 | 18.8 | 9.4 |
| LPA C18:1 | 3200.0 | 1600.0 | 800.0 | 400.0 | 200.0 | 100.0 | 50.0 | 25.0 | 12.5 |
| LPA C18:0 | 2666.7 | 1333.3 | 666.7 | 333.3 | 166.7 | 83.3 | 41.7 | 20.8 | 10.4 |
| cLPA C18:1 | 2400.0 | 1200.0 | 600.0 | 300.0 | 150.0 | 75.0 | 37.5 | 18.8 | 9.4 |
| S-1-P C18:1 | 8000.0 | 4000.0 | 2000.0 | 1000.0 | 500.0 | 250.0 | 125.0 | 62.5 | 31.3 |
| Sph C18:1 | 9600.0 | 4800.0 | 2400.0 | 1200.0 | 600.0 | 300.0 | 150.0 | 75.0 | 37.5 |
| Spha C18:0 | 9600.0 | 4800.0 | 2400.0 | 1200.0 | 600.0 | 300.0 | 150.0 | 75.0 | 37.5 |
| PAF C16:0 | 1333.3 | 666.7 | 333.3 | 166.7 | 83.3 | 41.7 | 20.8 | 10.4 | 5.2 |
